# The Direct Economic and Opportunity Costs of the Medical College Admissions Test (MCAT) for Canadian Medical Students

**DOI:** 10.15694/mep.2018.0000243.1

**Published:** 2018-10-25

**Authors:** Yannick Fortin, Kulamakan (Mahan) Kulasegaram, Jesse N. Kancir, Geneviève Moineau, Ariane Carpentier, Mark D. Hanson

**Affiliations:** 1 University of Ottawa; 2 The Wilson Centre; 3 School of Population and Public Health; 4 Association of Faculties of Medicine of Canada and the Department of Paediatrics at the University of Ottawa; 5 Faculté de Médicine; 6 Department of Psychiatry

**Keywords:** Admissions, Selection, Recruitment, Personal Characteristics, MCAT, Costs, Opportunity Cost, Direct Cost, Examination

## Abstract

This article was migrated. The article was marked as recommended.

Background

The Association of Faculties of Medicine of Canada, Future of Medical Education in Canada report shared a collective vision to improve social accountability, including a review of admissions policies to enhance student diversity. This study explored if and how the Medical College Admissions Test (MCAT) might mediate the socioeconomic diversity of Canadian medical schools by quantifying the costs and other cost-related factors of preparing for the exam.

Methods

A 34-question anonymous and bilingual (English and French) online questionnaire was sent to the 2015 first-year cohort of Canadian medical students. Developed collaboratively, the survey content focused on MCAT preparation and completion activities, associated costs, and students’ perceptions of MCAT costs.

Findings

The survey response rate was 32%. First-year medical students were more likely than the Canadian population to belong to high-income families (63% vs. 36%) and less likely to be from rural locations (4.5% vs. 19%). Use of MCAT preparation materials was reported by nearly every MCAT test-taker (95.3%): of those, 76.4% used free practice tests; 59.8% paid for practice tests; 45.1% registered for preparation courses; and 3.3% hired a private tutor. In terms of writing the MCAT, the total economic costs per respondent are estimated at $6,357 ($4,755-$7,958) and total direct costs per respondent are estimated at $2,970 ($1,882- $4,058). Opportunity costs represented the majority of economic costs, at $3,387 ($2,872 - $3,901), or 53.2%. MCAT preparation costs are estimated to be $2,372 ($1,373-$3,372), or 79.9% of total direct costs and 37.3% of economic costs. Most respondents agreed, 76%, that the MCAT posed a financial hardship.

Conclusion

The financial demands of preparing for and completing the MCAT quantified in this study highlight an admissions requirement that is likely contributing to the current student diversity challenges in Canadian medical schools. In the spirit of social accountability, perhaps it is time to prioritize equitable alternative for assessing applicants’ academic readiness for medical school.

## Introduction

The selection of medical students relies on assessments of academic readiness to ensure success during training. In North America, as in other jurisdictions, a popular approach is to use the grade point average and an achievement test. In Canada and the United States, many but not all medical schools use the Medical College Application Test (MCAT) as a selection tool for academic readiness ( Eskander, Shandling and Hanson, 2013). Previous evaluations of this test have demonstrated reasonable evidence of predictive validity in the US and Canadian contexts, typically against in-program academic performance indicators but also for licensing examination performance ( Siu and Reiter, 2009; Davis *et al.*, 2013). An additional advantage of the MCAT is that it bypasses systematic differences in grades due to institutional and degree variability ( Didier *et al.*, 2006).

However, student selection based on academic readiness is only one of several competing priorities for medical schools. In North America, admission committees, as well as national medical education organizations, are placing greater emphasis on the importance of expanding student diversity. This is a salient challenge in Canada where the Association of Faculties of Medicine of Canada (AFMC), the Committee on Accreditation of Canadian Medical Schools (CACMS), and individual schools have made diversity an important element in the agenda for change. With renewed attention on the role of admissions practices as a factor affecting diversity, admissions tools are being evaluated for their impact on expanding or reducing aspects of socio-demographic diversity. Tools that systematically exclude under-represented minorities or prove to be barriers to equity may not meet acceptability criteria despite other forms of validity evidence. One demographic group for which there has been active interest in expanding access to medical school are students from the lower-socioeconomic strata. Recent literature in medical education have addressed the issues of barriers for lower socioeconomic medical students and applicants before and during medical training ( Busing, Rosenfield and Rourke, 2010). Thus, activities at the admissions level to expand diversity have focused on socioeconomic diversity.

The role of the MCAT in impacting this diversity should therefore be evaluated in terms of economic costs and potential for impact upon applicant socio-economic diversity. In other jurisdictions, there is some evidence that standardized testing for admissions disadvantages applicants from lower socio-economic strata though data in North America and Canada specifically are not available ( Puddey and Mercer, 2013). Further, cost analysis of the MCAT and other admissions standardized tests have been identified as a direction for future research inquiry ( Patterson *et al.*, 2016). While ostensibly, the upfront costs of the MCAT and similar standardized tests are low, other factors increase the expense of the test. Of particular concern is that completion of the MCAT is associated with a culture of preparation that includes taking intensive training courses, foregoing employment opportunities in order to prepare, and making a significant commitment in time and money to complete the exam itself ( Eskander, Shandling and Hanson, 2013). In economic terms, the direct, indirect, and opportunity costs of the MCAT could be a significant challenge not only for applicants, but also admissions committees wishing to advance the socioeconomic diversity of their medical students.

The objective of this study is to explore if and how the MCAT might mediate the socio-economic diversity objectives of Canadian medical schools by quantifying the costs to MD students of preparation and completion, including the direct and opportunity costs of testing, and students’ perceptions of MCAT cost-related factors.

## Methods

### The MCAT

The MCAT evaluated for this study is the pre-2015 version, which is a standardized multiple-choice test which evaluates verbal reasoning, biological science, and physical science. Since 2015 the test format has changed and participants in this study would have completed the older version of the MCAT. The MCAT is a timed computerized test that must be completed at a designated test center. The cost to take the MCAT for members of the study cohort was U$275 dollars.

### Survey Development and Stakeholder Consultations

The absence of a national data repository on medical students’ MCAT completion and preparation activities motivated the development of the Pan-Canadian Survey of Medical Students’ Knowledge and Experiences Regarding Medical College Admission Test (MCAT) Completion and Preparation Activities (henceforth the survey). This initiative was accomplished in consultation with a broad array of medical education stakeholders, including: Canadian Undergraduate Medical Education (UGME) Deans, medical school admissions deans, directors, and administrative staff, the AFMC Distributed Medical Education (DME) executive group, Medical Admissions Committee, the Council of Ontario Faculties of Medicine (MAC-COFM), the University of Toronto’s Faculty of Medicine Undergraduate Medicine Executive (UME) Committee, and medical students via representation by the Canadian Federation of Medical Students (CFMS) and the Fédération médicale étudiante du Québec (FMEQ). This collaborative survey development process was managed centrally by the AFMC, which was also the survey data custodian. Ethical approval for this project was obtained from the University of Toronto Health Sciences Research Ethics Board. Informed consent was required from all respondents prior to survey initiation. The survey was anonymous and voluntary.

### Survey Content

A 34-question survey focused on socio-demographic indicators, pre-medical education, MCAT preparation and completion activities, associated costs, and students’ perceptions of MCAT costs was developed for online administration (see supplementary materials). The survey questions were developed with the aim of identifying the proportions and characteristics of Canadian first year medical students participating in MCAT completion and preparation activities, their patterns of use, and the associated financial costs of MCAT-related activities. In addition, the survey asked medical students about their perceptions of MCAT costs.

A preliminary version of the survey was distributed to stakeholders for evaluation and survey refinement. The respondent demographic questions were modeled on Statistics Canada’s 2011 Census of Population to allow for comparisons with the general Canadian population. Pilot testing, which evaluated the qualitative and technical dimensions of the survey, was performed by survey team members, medical student volunteers, and AFMC staff.

### Population, Survey Frame and Distribution Strategy

The survey frame consisted of the mailing lists of the medical student associations at each of the participating medical schools and survey distribution was facilitated by the two Canadian national medical student associations (CFMS and FMEQ). The target population was the 2,911 first-year medical student cohort of the 2015/2016 class. We elected to conduct a census of first-year medical students for reasons of feasibility and acceptability: medical students were accessible to the study team and could be surveyed readily with institutional approvals. Access to medical school applicants who were not successful in being selected to medical school was administratively and financially prohibitive.

The initial survey invitation and reminder emails included a survey project description, consenting information, and an embedded survey URL. A total of four survey reminders were sent to potential respondents during the data collection period which lasted from September 4, 2015 to October 31, 2015. Survey data was collected using the Canadian-based Fluid Surveys online survey platform.

### Cost Analysis

**Figure 1.**
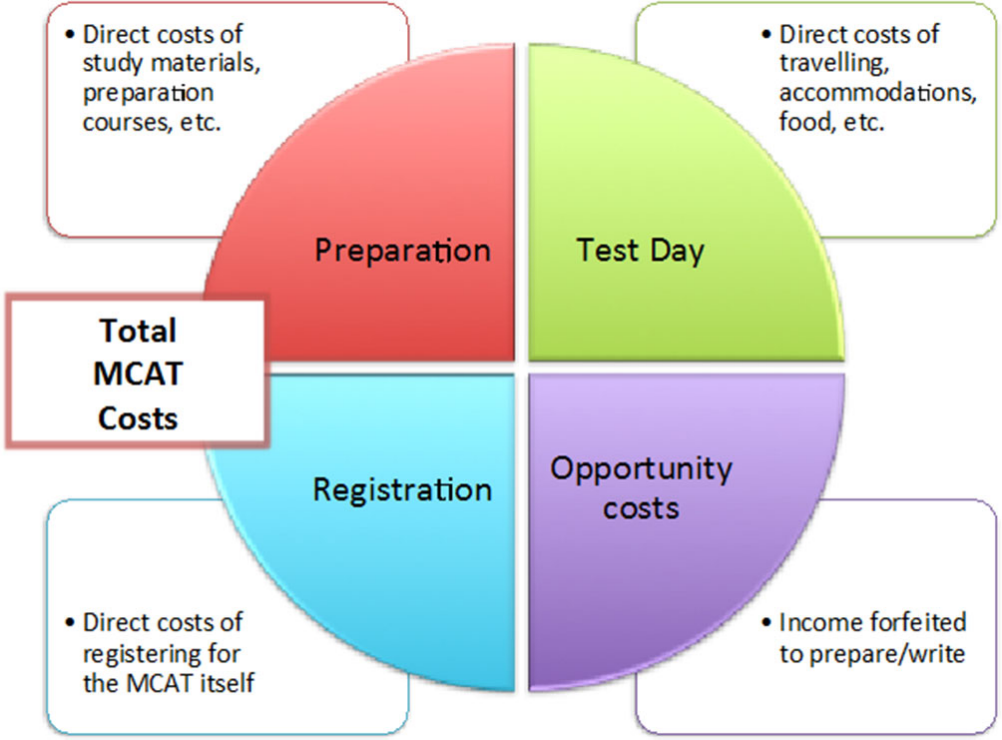
Direct and Opportunity Costs of the MCAT

Three types of costs were considered in this analysis: direct, indirect, and opportunity costs ( Walsh, 2014) ( Figure 1). Direct costs are those incurred specifically by a single activity, which for this study would include MCAT registration costs, preparation materials, and costs incurred on the day of the test. Indirect costs are those shared across multiple activities, such as accommodation and food costs incurred while preparing for the test and are theoretically apportioned across activities. Opportunity costs are those that measure the value of what has been forfeited in pursuing an activity, which for the purpose of this study represents the amount of income foregone as a result of preparing for and taking the MCAT.

Given the complexity of accurately calculating indirect costs in a survey format, the decision was made to exclude them from the survey. Other specific economic analyses were not possible as widely accepted utility and effectiveness outcome measures are not available or vary greatly. Nevertheless, by combining the direct and opportunity costs, this study estimated an economic cost which captured the true impact on applicants more fully. Cost data was collected using qualitative variables to simplify data gathering, and cost categories were estimated using the category midpoints, though ranges are provided using both the low- and high-ends of the response categories.

### Data Analysis

Categorical variables are described by frequency distributions and compared using the Pearson Chi-square (χ2) test. Continuous variables are presented with means and compared with Student’s t-test. For a subset of parameters, bivariate logistic regression was used to derive odds ratios. Statistical significance was established based on p values ≤ 0.05. Analyses were performed using SAS version 9.4 (SAS Institute Inc, Cary, NC).

## Results/Analysis

### Respondent Characteristics

The survey was completed by 922 of the 2,911 targeted respondents, for a response rate of 32%. Response rates across the medical schools ranged from 13.0% to 57.9%. Results of the non-parametric Satterthwaite method demonstrated that response rates from medical schools using the MCAT as an admission requirement did not differ significantly from those that do not have that requirement. The mean response rate of MCAT schools was 31.9% while the mean response rate of non-MCAT schools was 31.4%, p=0.92.

The characteristics of respondents are presented in table 1. The majority of respondents, 58%, were female. This figure is slightly above and statistically different than the proportion of first-year female medical students (53%) in 2015-16, χ2 (1, N = 922) = 6.505, p<.01 ( AFMC, 2016). The median age of respondents was 23 years and 76% had achieved a bachelor’s degree prior to entry to medical school. Of note, for this study respondents were mostly from high-income families; with 62.6% reporting that the combined annual income of their parents was greater than $100,000; only 7% of respondents were from families earning less than $40,000 annually. A large proportion of respondents had at least one parent that had achieved a graduate or professional university degree (47%); with 8% of respondents having parents whose highest education was high school. Nearly one third of respondents, 35%, had an immediate family member employed as a health care professional.

**Table 1.** Respondent Characteristics, N=922

Variable	Count (%) ^1^
**Sex**	
Female	530 (57.9)
Male	385 (42.1)
*Missing*	*7*
**Age (years)**	
Mean	23,4
Below 20	57 (6.4)
20 - 22	351 (39.3)
23 - 25	341 (38.2)
26 - 30	99 (11.1)
Over 30	45 (5.0)
*Missing*	*29*
**Race/ethnicity**	
Arab	24 (2.6)
Asian	100 (10.9)
Black	11 (1.2)
Caucasian	579 (63.5)
Chinese	96 (10.5)
Filipino	6 (0.7)
Latin American	5 (0.6)
North American Aboriginal	5 (0.6)
Other, incl. mixed	86 (9.4)
*Missing*	*10*
**Educational Attainment** ^2^	
CEGEP	132 (14.4)
Some Undergrad	90 (9.8)
Bachelors’ Degree	506 (55.1)
Some Graduate	29 (3.2)
Master’s Degree	122 (13.3)
Doctorate Degree	27 (2.9)
Other	12 (1.31)
*Missing*	*4*
**Language**	
Unilingual English	497 (54.1)
Unilingual French	76 (8.3)
Bilingual Eng/Fr	247 (26.9)
Bilingual Eng or Fr / Other	64 (7.0)
Trilingual Eng/Fr/Other	34 (3.7)
*Missing*	*4*
**Community Size During High School** ^3^	
*Below 1,000*	30 (4.5)
*Between 1,000 and 9,999*	104 (15.4)
*Between 10,000 and 29,999*	86 (12.8)
*Between 30,000 and 99,999*	141 (20.9)
*Between 100,000 and 1,000,000*	312 (46.4)
*Missing*	*249*
**MCAT Completion**	
Yes	673 (76.8)
No	203 (23.2)
Missing	46

Abbreviations: Eng, English; Fr, French

^1^ Percentages exclude missing values.

^2^ Highest level of education at the start of medical school.

^3^ Size of population during high school or equivalent studies.

### MCAT Completion Preparation Activities and Associated Costs

Of the students surveyed, the MCAT was completed by 76.8% of responders. Of the 365 responders admitted to medical schools without MCAT requirements, 41.5% had completed the MCAT regardless. The MCAT exam can be completed repeatedly. In our total sample, 54.2% of respondents had completed it once, 29.4% completed it twice, and 16.4% completed it three or more times. Use of MCAT preparation materials was reported by nearly every MCAT test-taker (95,3%): of those, 76.4% used free practice tests; 59.8% paid for practice tests; 45.1% registered for preparation courses; and 3.3% hired a private tutor.

### Direct Cost: Registration

Given that the cost of the MCAT is in US dollars, to account for differing US registration costs and exchange rates across time we used the 2014 cost of application of $275 USD with no currency conversion as a standard cost for all attempts. This base cost assumption of $275 CAD was then multiplied by the attempt frequency, with possible ranges of 0-5 attempts (where 5 represented five or more attempts). Average registration costs across the sample were $355 per student (see supplementary materials, Table A).

### Direct and Indirect Preparation Costs

Preparation costs were subdivided into two categories: costs of preparatory courses, and all other materials. This was done to separately analyze the assumedly disproportionately high cost of courses (see supplementary materials, Tables B and C).

Substantial costs were associated with MCAT preparation, which were estimated at $2,372 ($1,373-$3,372) per respondent. The individual cost of preparatory courses was estimated at $1,731 ($1,232-$2,231), and the individual costs of non-preparatory course material were $641 ($141 -$1141). Most course completers (62.9%) spent between $1,001 and $2,000 on preparation courses. Of the MCAT completers who did not participate in a preparation course, 82.1% cited that cost as prohibitive. Having to work full-time was cited as a reason to forego a preparation course by 33.8% of these respondents.

In 52% of cases, parents and family members paid or helped to pay for MCAT preparation activities. Respondents from families in the highest income bracket were more likely to participate in paid MCAT preparation activities. Respondents whose parents earned more than $100,000 annually were 54% more likely than those whose parents earned less than $100,000 annually to have completed a preparation course (OR = 1.54, 95% CI: 1.10-2.17, p= 0.0126).

### Test Day Costs

Costs incurred on the day of testing, for transportation and accommodations, were small for 73% of completers at $250 or less but exceeded $1,000 for 5% of respondents. Overall, the individual test day costs were estimated to be $243 ($155-$331) (see supplementary materials, Table D).

### Opportunity Costs

When asked if preparing for the MCAT affected their ability to earn income, 35% of respondents acknowledged they reduced their working hours while another 30% did not take a job A greater percentage of respondents who reduced their working hours (56.4% vs. 43.6%) or did not take a job (68.2% vs. 31.8%) to study for the MCAT had parents with earnings in the highest income bracket ($100,000 or more vs. less than $100,000 annually), respectively. Overall, respondents reported average opportunity costs of doing the MCAT of $3,387 ($2,872 - $3,901), representing the single highest economic cost of the analysis. (see supplementary materials, Table E)

### Total Costs

The total economic costs (i.e. the sum of the direct and opportunity costs) of the MCAT per respondent are reported in Table 2. The estimated average economic cost was $6,357 ($4,755 - $7,958). The direct costs (registration, preparation, test taking) was estimated to be $2,970 ($1,882- $4,058). As a proportion of total costs, MCAT registration represented 11.9% (8.7% - 18.9%) of the estimated direct costs (including preparatory courses), and 5.6% (4.5% - 7.5%) of estimated economic cost.

**Table 2.** Total Direct and Economics Costs of MCAT-Related Completion and Preparation Activities

Fee type	Average Cost	Low Range	High Range
**Direct Costs**			
MCAT Test Registration, N=876	$355	$355	$355
Preparation Materials, N=609	$641	$141	$1,141
Test Day, N=629	$243	$155	$331
Preparation Course, N=299	$1,731	$1,232	$2,231
**Economic Costs**			
Opportunity Cost, Lost Wages, N=514	$3,387	$2,872	$3,901
**Total Costs**			
Total, All	$6,357	$4,755	$7,958
Total, No Prep Course	$4,625	$3,523	$5,728
Total, Direct Costs Only w/ Course	$2,970	$1,882	$4,058
Total, Direct Costs Only w/o Course	$1,239	$651	$1,827

### Opinion of the Value of the MCAT

Respondents were asked about the financial implications of having to complete the MCAT: 76% agreed that MCAT costs represent a financial hardship in the admission process.

## Discussion

This study aimed to document the direct and opportunity costs to medical school students of taking the MCAT in Canada in order to analyze potential effects on socio-economic diversity. Our data show that MCAT costs go beyond the registration cost for the test day and include substantial direct and opportunity costs for the successful medical schools. This cost even extended to the population of students in medical schools that did not require the MCAT.

The most obvious and direct costs for the MCAT are test registration and preparation materials. The use of MCAT preparation materials is ubiquitous within this population of medical students and associated financial costs of the MCAT although substantial are not evenly distributed across the arc of MCAT preparation and completion. For instance, MCAT registration and test day transportation and accommodations costs represent a smaller proportion of total costs in comparison to costs associated with MCAT preparation activities. However, other hidden costs such as transportation and accommodation for the test also play a factor. For applicants from rural settings and thus further away from test centres, these costs are likely to much higher than for students from urban or suburban settings. Another “hidden” cost is the high opportunity cost of MCAT preparation activities which constitutes the largest proportion of total preparation costs. Intensive preparation for the MCAT meant a meaningful proportion of our sample opted out of other economic activities.

Recognizing the high cost of the MCAT for medical school applicants is only the beginning. The study results further show that MCAT preparation and completion is commonly shared amongst family members, with 52% of students reporting that parents and family members paid or helped to pay for MCAT-related expenditures. Our results suggest that not all families can share this financial burden equally. Medical student participation in certain paid MCAT preparation activities was associated with parental income, with students whose parents are high income earners being more likely to participate in MCAT preparation courses. In addition, many medical students decreased their work hours or did not take on employment to participate in MCAT preparation activities. Of those who implemented such options, students hailing from higher earning families were overrepresented compared to those whose families had lower means. As expected, the socioeconomic profile of our survey respondents revealed an overrepresentation of higher income families when compared to the general population. While 62.6% of study respondents reported a combined parental income above $100,000 annually, only 37.1% of couple and lone parent families in the country were in that same income category in 2014, according to Statistics Canada (2017).

Recently, the medical school admissions and selection literature has begun to critically evaluate the role of standardized testing in light of social and political accountability goals ( Kelly *et al.*, 2014; Kelly and O’Flynn, 2017; Kumar *et al.*, 2018). In particular, addressing socio-economic diversity has become a concern for many education contexts including North America and Europe ( Southgate, Kelly and Symonds, 2015; Brosnan *et al.*, 2016). At the same time, there is an increasing call for using cost as an outcome to understand and evaluate medical education practices ( Walsh *et al.*, 2013; Zendejas *et al.*, 2013; Tolsgaard and Cook, 2017). To our knowledge, this is the first Pan-Canadian study to administer a census on the participation of medical students in MCAT completion and preparation activities and publish costs estimates; albeit self reported. These data are important as they provide evidence on the financial burden of taking the MCAT and the distribution of that burden across the spectrum of medical students. Thus, it has direct relevance to efforts to address the socioeconomic diversity of medical students. Our data supports initiatives such as those undertaken by the AFMC to pilot a MCAT fee assistance program for Canadian MCAT test-takers with identified financial need. This financial assistance is currently directed towards Canadian MCAT registration costs. This coupled with the availability of free MCAT preparation materials is a laudable first step in addressing student socioeconomic diversity but our data suggests more will be necessary.

As our data show, the cost of the MCAT is still significant and extends to students who were accepted to schools that do not require it during the application process. In the admissions vernacular of Canada, medical schools are categorized as MCAT and Non-MCAT schools. This categorization may lead one to believe that the aforementioned economic burden is largely limited to medical students and their families attending MCAT schools. Yet, 41.5% of survey respondents registered at Non-MCAT schools had completed the MCAT. This observation is likely reflective of Canada’s highly competitive admissions environment which incentivises applicants to apply across schools with different MCAT requirements. This raises an important question about whether effective changes to admission requirements should focus on individual schools or reflect national priorities. While admission requirements vary to serve specific geographic and associated medical student diversity goals, the possible socioeconomic consequences of relying upon the MCAT may ultimately represent a key challenge to the socioeconomic diversity of the medical student body from a national perspective.

Our study has limitations. First, our response rate was 32%, a figure that may appear low. However, this rate mirrors that of the National Physician Survey in Canada, which includes an e-mail-based survey of medical students. For these students, the national response rate was 30.8% in 2007 and 31.2% in 2004 ( Grava-Gubins and Scott, 2008). Moreover, the demographic characteristics of our study’s respondents parallels published reports from other Canadian medical student studies ( Dhalla *et al.*, 2002; Young *et al.*, 2012). It must also be acknowledged that women respondents were overrepresented compared to men in this study, which implies that differential approaches to MCAT preparation and completion activities by gender, if any, could result in some differences in the cost estimates reported.

Second, we surveyed for the “old” MCAT preparation and completion activities prior to implementation of the new MCAT 2015 ( AAMC MCAT Validity Committee, 2017). For this latest version of the exam, the Association of American Medical Colleges (AAMC) has made an effort to level the cost and accessibility barriers for adequate preparation. For instance, the AAMC collaborated with the Khan Academy to enhance accessibility to free preparation materials for under-represented student groups. The availability of free and lower cost preparation materials, however, may have a paradoxical impact within the applicant pool. Those applicants with readily available social and financial capital may be able to leverage their personal, family and social resources in order to purchase a range of more expensive preparation course materials while simultaneously availing themselves of the lower cost materials. In contrast, applicants without such resources will be restricted to participate in the lower cost end of the MCAT preparation market. Of course, one will have to await the evaluation of the impact of new approaches such as the free online preparation materials. Our data set therefore represents a baseline cost measure for such future cost comparisons across “old” MCAT preparation costs with the new MCAT 2015 course preparation costs.

Third, our study was limited to the Canadian MCAT course preparation marketplace and our findings may realistically represent only the tip of the financial iceberg underpinning the global MCAT course preparation marketplace. The MCAT is used for application to schools in the US, Caribbean, and other jurisdictions. Moreover, the proliferation of standardized tests in other jurisdictions raises similar issues of cost and preparation.

Fourth, our findings must be considered in light of the fact that we only surveyed medical students who were accepted into medical programs. For each of these students, there are numerous others who invested in MCAT preparation and completion activities but who will never recoup this investment while employed as physicians. Important questions remain regarding applicants who were not admitted to medical school. For instance, what is their diversity profile and does it differ from those entering MD programs? Likewise, should our focus for those applicants from underrepresented student groups be upon access to affordable MCAT preparation activities or upon the social determinants of pre-medical educational attainment? Exploring these issues will require a broader survey of applicants and objective evaluation of the efficacy of preparatory activities.

## Conclusion

Our study examined the direct economic and opportunity costs of MCAT preparation and completion for Canadian medical students as well as their perceptions of MCAT costs. The MCAT represents a financial burden for medical students that is especially problematic given efforts to reach applicants from lower socio-economic groups and to expand access to medical training. In light of continuing student diversity challenges in Canadian medical schools and the arguably prohibitive financial demands of MCAT preparation and completion activities, it appears it is time to consider more equitable alternatives for assessing applicants’ academic readiness for medical school.

## Take Home Messages

• The total economic costs of preparing and completing the MCAT was estimated to exceed $6,300 CAD for first-year Canadian medical students.
• Opportunity costs represented the majority of economic costs, 53.2%, associated with preparing and completing the MCAT.
• A large majority of medical students, 76%, agreed that MCAT costs represent a financial hardship in the admission process.
• MCAT completers from highest-income families were more likely than those with lower means to participate in paid MCAT preparation courses, and to reduce their working hours or not take a job in order to study for the exam.
• Canadian first-year medical students remain more likely to belong to high-income families and to originate from urban locations than the Canadian general population.

## Notes On Contributors

Dr. Yannick Fortin is a population health researcher and former Director of Data & Analysis for the Association of Faculties of Medicine of Canada (AFMC). ORCID: 0000-0001-7300-9549

Dr. Kulamakan (Mahan) Kulasegaram is a Scientist at the Wilson Centre, Assistant Professor in the Department of Family & Community Medicine and Scientist at the Centre for Ambulatory Care Education, Women’s College Hospital at University of Toronto. ORCID: 0000-0002-6644-0098

Dr. Jesse N. Kancir is a resident physician in public health at the University of British Columbia, he is past President of the Canadian Federation of Medical Students and is on the boards of several national medical organizations.

Dr. Geneviève Moineau is President and CEO of the Association of Faculties of Medicine of Canada (AFMC). She is an Associate Professor in the Department of Paediatrics at the University of Ottawa where practices Pediatric Emergency Medicine at the Children’s Hospital of Eastern Ontario.

Dr. Ariane Veilleux Carpentier is a resident physician in neurology at the Centre hospitalier de l’Université de Montréal (CHUM), she is the past president of the Fédération médicale étudiante du Québec (FMEQ).

Dr. Mark D. Hanson is a Child and Adolescent Psychiatrist at the Hospital for Sick Children and is Professor of Psychiatry at the University of Toronto. He is the Past MD Admissions and Student Finances Associate Dean/Director at the University of Toronto Faculty of Medicine. ORCID: 0000-0002-0820-4521

## Declarations

Dr. Mark D. Hanson is a Co-Theme Editor for the theme of Selection and Recruitment in Medical Education in AMEE MedEdPublish.
