# Supplementary material for: The Direct Economic and Opportunity Costs of the Medical College Admissions Test (MCAT) for Canadian Medical Students
Source: MedEdPublish (2016). 2018 Oct 25;7:243. [Version 1] doi: 10.15694/mep.2018.0000243.1 (PMC10711961; doi:10.15694/mep.2018.0000243.1)
Supplement: Peer review history

# Reviewer report: Reviewer response for version 1

**DOI:** 10.21956/mep.19691.r29433

**Published:** 2019-07-23

**Authors:** Gary D. Rogers

**Affiliations:** 1 Griffith University

This review has been migrated. The reviewer awarded 5 stars out of 5 Thank you, colleagues, for this well-written paper reporting what appears to have been a carefully-executed and important study. The methodology has clear limitations, especially those related to the ‘missing’ population who were not recruited to medical school, but these were pragmatically unavoidable and are fully acknowledged by the authors. The insights obtained nonetheless remain very important. They underline the fact that in most developed countries we continue, de facto, to select for entry to medical school primarily on the variable of parental income. This is a scandalous state of affairs that requires radical action. Given that many medical schools acknowledge that the use of aptitude tests is primarily a tool to reduce the volume of applicants prior to other assessments such as multiple mini interview, and that undergraduate GPA appears to be a better predictor of performance in (postgraduate) medical programs, this study underlines that their ongoing use is verging on the unconscionable. Other, fairer, means for ‘numbers reduction’ must be sought and the reintroduction of lottery-like systems, utilised formerly in the Netherlands, needs to be reconsidered.

Reviewer Expertise:

NA

No decision status is available

# Reviewer report: Reviewer response for version 1

**DOI:** 10.21956/mep.19691.r29435

**Published:** 2018-11-12

**Authors:** Tonya L. Fancher

**Affiliations:** 1 UC Davis School of Medicine, General Medicine, Geriatrics, and Bioethics

This review has been migrated. The reviewer awarded 4 stars out of 5 Thank you for inviting me to review this paper.This important work contributes to the body of knowledge on how our medical education system must re-assess the unintended consequences and structural barriers to ensuring a diverse healthcare workforce. It is time for us to create a system of educational equity for all -- and support students from lower income families to become the physicians that our communities need.

Reviewer Expertise:

NA

No decision status is available

# Reviewer report: Reviewer response for version 1

**DOI:** 10.21956/mep.19691.r29434

**Published:** 2018-11-02

**Authors:** Kieran Walsh

**Affiliations:** 1 BMJ

This review has been migrated. The reviewer awarded 4 stars out of 5 This is a good study. I have heard lots of discussions on widening participation in medicine but rarely is the issue of cost brought up. So it is great that more people are looking at this issue. There is sometimes mention of long-term student debt - but this study looks at much more immediate costs. This is practical problem for candidates and their families. In the future cost should be part of our thinking in all aspects of medical education - including selection. (Maloney et al 2017). The authors are correct in stating that an even greater issue may be those who did not succeed in their application and so who might have had a significant outlay with no return. This group should also be looked at. Maloney S, Reeves S, Rivers G, Ilic D, Foo J, Walsh K. The Prato Statement on cost and value in professional and interprofessional education. J Interprof Care. 2017 Jan;31(1):1-4.

Reviewer Expertise:

NA

No decision status is available

# Reviewer report: Reviewer response for version 1

**DOI:** 10.21956/mep.19691.r29431

**Published:** 2018-11-02

**Authors:** Barbara Griffin

**Affiliations:** 1 Macquarie University

This review has been migrated. The reviewer awarded 4 stars out of 5 In providing a basic analysis of the costs involved for medical school applicants to sit one of the common entry tests of cognitive ability, this paper presents evidence that speaks to the global issue of widening participation in medicine. In many cases, standardised tests such as the MCAT were introduced, at least in part, to create a ‘level playing field’ on which to compare applicants who come from a wide range of educational backgrounds. However, an unintended consequence of using such tests is that they become a magnet for the commercial coaching industry. Indeed, Fortin et al. show how the majority of those who were successful in gaining a place in a Canadian medical school had felt the need to pay a not insignificant amount of money for commercial test preparation material and coaching. Of concern, survey respondents indicated that the use of the MCAT therefore created financial hardship, many relying on family and friends for help with costs. As the authors conclude, the lack of economic diversity among medical students might be exacerbated by using the MCAT to make selection decisions.Although the data relate to an older version of the MCAT, it is unlikely the newer format would present less of a financial cost. However, even though the authors achieved their aim of quantifying costs, there remains further questions before they can assess whether use of the MCAT “might mediate the socioeconomic diversity of Canadian medical schools”. For example, do those from low SES have an idea of the costs involved and does this knowledge dissuade them from applying? Did the authors collect MCAT results? If so, it would be interesting to know if performance was related to money spent. The authors offer some comment on overcoming the potential impact for low socioeconomic status applicants but appear to assume that the involvement of commercial coaching is inevitable. Given that coaching might threaten test reliability and validity (Griffin, 2018), there needs to be a more concerted response to the issues.Griffin, B. (2018). Coaching issues. In F. Patterson & L. Zibarras (Eds.) Selection and Recruitment in the Healthcare professions: research, theory and practice. London: Palgrave McMillan.

Reviewer Expertise:

NA

No decision status is available

# Reviewer report: Reviewer response for version 1

**DOI:** 10.21956/mep.19691.r29429

**Published:** 2018-10-25

**Authors:** Trevor Gibbs

**Affiliations:** 1 AMEE

This review has been migrated. The reviewer awarded 5 stars out of 5 Thank you for asking me to review this paper. I enjoyed reading it very much and learned quite a lot from it.It was a shame about the low response rate but I am not sure that it affected the results and itself can be the subject of a research paper of why this low response rate.I feel that it is very important to look at the cost of education when we set different aspects of admissions; sadly we do not very often, so I feel that this paper is an essential read for all involved in curriculum planning and admission procedures. There is much to discuss and debate from this paper and it would be very interesting to see how the Canadian schools respond either individually or as a National group. Well done

Reviewer Expertise:

NA

No decision status is available

# Reviewer report: Reviewer response for version 1

**DOI:** 10.21956/mep.19691.r29432

**Published:** 2018-10-25

**Authors:** Felix Silwimba

**Affiliations:** 1 University of Lusaka

This review has been migrated. The reviewer awarded 5 stars out of 5 This is a thought-provoking study for a medical educator from a low- or middle-income country. It is informative well written and organised. The findings are relevant. Although low- and middle-income countries still suffer from the dilemma of socialized medical care and training of medical personnel to an extent that classes are not clearly visible or openly talked about. My interest is even in this country the private sector is gradually getting involved in medical training. The question is how this will affect equity in medical school enrollments. The other issue is academic dishonesty is rampant in low- and middle-income countries at the level of secondary school education. The introduction of medical school examination can be a solution. This study has enlightened me very much.

Reviewer Expertise:

NA

No decision status is available

# Reviewer report: Reviewer response for version 1

**DOI:** 10.21956/mep.19691.r29430

**Published:** 2018-10-25

**Authors:** Jennifer Cleland

**Affiliations:** 1 University of Aberdeen

This review has been migrated. The reviewer awarded 4 stars out of 5 Good to see a paper looking at the cost of selection given the increasing calls to look at the cost and value of medical education. The cost analysis in this paper is quite simplistic but appropriate, but those wishing to know more about how to look at cost and value in medical education may be interested in a forthcoming AMEE guide on just this topic (lead author Jonothan Foo). There is also a paper in early view in Medical Education looking at the cost of selection from another point of view, that of the medical school.

Reviewer Expertise:

NA

No decision status is available
